# Molecular markers of antifolate resistance in *Plasmodium falciparum *isolates from Luanda, Angola

**DOI:** 10.1186/1475-2875-10-248

**Published:** 2011-08-24

**Authors:** Bianca E Gama, Guilhermina AL Pereira-Carvalho, Florbela JI Lutucuta Kosi, Natália K Almeida de Oliveira, Filomeno Fortes, Philip J Rosenthal, Virgílio E do Rosário, Cláudio Tadeu Daniel-Ribeiro, Maria de Fátima Ferreira-da-Cruz

**Affiliations:** 1 Laboratory of Malaria Research, Instituto Oswaldo Cruz, Fiocruz, Rio de Janeiro, Brazil; 2 Health Progress and Investigation Network of the Portuguese-Speaking Countries Community (RIDES/CPLP), Centro de Malária e Doenças Tropicais, Instituto de Higiene e Medicina Tropical, Universidade Nova de Lisboa, Lisboa, Portugal; 3 National Institute of Public Health, Luanda, Angola; 4 Department of Biology, Universidade Agostinho Neto, Luanda, Angola; 5 Department of Medicine, San Francisco General Hospital, University of California San Francisco, San Francisco, USA

## Abstract

**Background:**

*Plasmodium falciparum *malaria remains a leading health problem in Africa and its control is seriously challenged by drug resistance. Although resistance to the sulphadoxine-pyrimethamine (SP) is widespread, this combination remains an important component of malaria control programmes as intermittent preventive therapy (IPT) for pregnant women and children. In Angola, resistance patterns have been poorly characterized, and IPT has been employed for pregnant women since 2006. The aim of this study was to assess the prevalence of key antifolate resistance mediating polymorphisms in the *pfdhfr *and *pfdhps *genes in *P. falciparum *samples from Angola.

**Methods:**

*Plasmodium falciparum *samples collected in Luanda, in 2007, were genotyped by amplification and DNA forward and reverse sequencing of the *pfdhfr *and *pfdhps *genes.

**Results:**

The most prevalent polymorphisms identified were *pfdhfr *108N (100%), 51I (93%), 59R (57%) and *pfdhps *437G (93%). Resistance-mediating polymorphisms in *pfdhps *less commonly observed in West Africa were also identified (540E in 10%, 581G in 7% of samples).

**Conclusion:**

This study documents an important prevalence of 4 *P. falciparum *polymorphisms that predicts an antifolate resistance in Luanda. Further, some samples presented additional mutations associated to high-level resistance. These results suggest that the use of SP for IPT may no longer be warranted in Angola.

## Background

Sub-Saharan countries remain affected by *Plasmodium falciparum *malaria, with few exceptions. In 2009, there were an estimated 225 million cases of malaria and 781,000 deaths from malaria worldwide, most of them from Africa [1]. As in other parts of this continent, malaria remains a major problem in Angola, where the entire population is at risk of infection. In 2009, 3,726,606 suspected malaria cases were reported and 1,5734,222 malaria cases were confirmed by either RDT or microscopy. The number of confirmed outpatient cases remained nearly one million per year in the last four years, whereas inpatient malaria cases and deaths in 2009 decreased by about 31% and 13% respectively, compared to the annual average in 2000-2005. However, it is not clear from the available data whether this reflects a true decrease [1].

The control of malaria is seriously challenged by drug resistance. Resistance to the combination antifolate sulphadoxine-pyrimethamine (SP) is widespread, and this drug is no longer recommended for therapy of falciparum malaria [2]. However, SP resistance is at an intermediate level (e.g. parasite populations more tolerant to the drug but still cleared by the treatment) in most of Africa, and the drug remains an important component of malaria control programmes for use as intermittent preventive therapy (IPT) in pregnant women and children [3]. IPT involves periodic administration of full treatment doses of SP, regardless of the presence of parasitaemia [4]. IPT with SP has been efficacious in the prevention of malaria in pregnant women [5] and children [6], although protective regimens have not been optimized, and the impact of different levels of SP resistance on protective efficacy is not well understood [7].

Resistance to SP is mediated by point mutations in genes encoding the target enzymes dihydrofolate reductase (*pfdhfr*) and dihydropteroate synthetase (*pfdhps*) [8,9]. Increasing numbers of a well-defined set of mutations leads to increasing resistance [10,11]. In Africa, three *pfdhfr *(51I, 59R, 108N) and two *pfdhps *(437G, 540E) mutations are common in some areas, and the prevalence of the key polymorphisms, *pfdhfr *59R and *pfdhps *540E is associated with the treatment efficacy of SP [12,13]. The treatment efficacy of SP in IPT remains quite good in parts of west and central Africa, in large part due to the lack of one key polymorphism, *pfdhps *540E, in infecting parasites [14,15]. Considering differences in prevalence of this key polymorphism across Africa and its importance, the WHO recommends IPT with SP for infants only in areas with a prevalence of *pfdhps *540E < 50% [16]. Since IPT continues to be recommended in pregnancy across Africa, the aim of this study was to assess the prevalence of key antifolate resistance mediating polymorphisms in the capital of Angola, Luanda, where molecular resistance patterns have not been characterized yet, and where SP for pregnancy has been employed since 2006 [17].

## Methods

### Study site and population

Blood samples were collected in 2007 from outpatients attending for malaria diagnosis at four Luanda health facility posts located at the municipalities of Sambizanga, Imgombotas, Cazenga and Viana. Luanda has a population of approximately 4.5 million and is considered mesoendemic for malaria. Malaria is present throughout the year, with a marked increase in incidence after rains that peak during April and May.

Patients diagnosed with falciparum malaria based on Giemsa-stained thick smears were included in this study after informed consent was obtained. Inclusion criteria comprised individuals with age ≥ 12 years, no clinical evidence of complicated malaria and monoinfection with *P. falciparum*. The study was approved by the National Institute of Public Health/Angola Ethics Research Committee with agreement of the Instituto Oswaldo Cruz/Fiocruz.

### Study procedures

After enrollment, 5 ml of blood was collected by venipuncture and placed into ethylenediaminetetraacetic acid vacutainer tubes (Becton Dickinson). The samples were centrifuged (350 *g*, 10 minutes), and pellets were frozen with an equal volume of cryopreservation solution (0.9% sodium chloride, 4.2% sorbitol and 28% glycerol). Frozen samples were transported to the Instituto Oswaldo Cruz (Fiocruz), Rio de Janeiro, Brazil for further studies.

### DNA preparation

Blood samples were thawed, and 1 ml was used for DNA extraction with the QIAamp midi kit, as described by the manufacturer (Qiagen). Samples were resuspended in a final volume of 50 μl.

### Nested PCRs for *pfdhfr *and *pfdhps*

The *pfdhfr *and *pfdhps *amplification protocols were as described elsewhere [18]. Briefly, 2 μl of DNA solution was added to a 48 μl mixture containing 0.25 μM primers M1 (5' TTT ATG ATG GAA CAA GTC TGC 3') and M7 (5' CTA GTA TAT ACA TCG CTA ACA 3') for *pfdhfr *or primers N1 (5' GAT TCT TTT TCA GAT GGA GG 3') and N2 (5' TTC CTC ATG TAA TTC ATC TGA 3') for *pfdhps*. In the nested PCR, 2 μl of the initial PCR product was mixed with 0.25 μM primers M3b (5' TGA TGG AAC AAG TCT GCG ACG TT 3') and M9 (5' CTG GAA AAA ATA BCAT CAC ATT CAT ATG 3') for *pfdhfr *or primers R2 (5' AAC CTA AAC GTG CTG TTC AA 3') and R (5' AAT TGT GTG ATT TGT CCA CAA 3') for *pfdhps*. The *pfdhfr *primers amplify a 594 bp fragment comprising the SNPs A16V/S, C50R, N51I, C59R, S108N, V140L and I164L, and *pfdhps *primers amplify a 711 bp region containing SNPs S436A/F/C, A437G, K540E, A581G and A613T/S. PCR reactions were run in the GeneAmp PCR System 9700 (Applied Biosystems). Positive (DNA extracted from blood from patients with known *P. falciparum *infection) and negative (no DNA and DNA extracted from individuals who had never traveled to malaria-endemic areas) controls were also used in each round of amplification.

### PCR analysis and product purification

PCR products were separated by 2% agarose-gel electrophoresis. Products were purified through the Wizard SV Gel and PCR Clean-Up System (Promega), according to the manufacturer's instructions.

### DNA sequencing

DNA sequencing from forward and reverse strands was performed using *pfdhfr *or *pfdhps *nested PCR primers plus the purified product according to Big Dye^® ^Terminator Cycle Sequencing Ready Reaction version 3.1 instructions (Applied Biosystems). Sequences were read using an ABI PRISM DNA Analyzer 3730 (Applied Biosystems) from the Genomic Platform/PDTIS/Fiocruz [19]. Forward and reverse sequences were analysed using the free software Bioedit Sequence Alignment Editor version 7.0.5.2.

## Results

Samples were analysed from 66 patients, aged 21 to 30 years, with a diagnosis of acute uncomplicated falciparum malaria. Parasitaemia ranged from 500 to 100,000 parasites/μl. The *pfdhfr *analysis yielded 61 sequences and that for *pfdhps *30 sequences. The failure to satisfactory amplify *pfdhps *gene in Angolan samples might be somehow attributed to primer limitations due to unknown polymorphisms in target sequences.

The most prevalent polymorphisms were *pfdhfr *108N (100%), 51I (93%), 59R (57%) and *pfdhps *437G (93%) (Table 1). In addition, other polymorphisms were detected, including 50R in the *pfdhfr *gene (6%) and 540E (10%) and 581G (7%) in the *pfdhps *gene. No mutations were found at 16, 140 and 164 codons from *pfdhfr *gene. Considering haplotypes, the most prevalent were CIRN (51%) and CICN (34%) for *pfdhfr *gene (codons 50, 51, 59 and 108), and SGKAA (60%) and AGKAA (23%) for *pfdhps *gene (codons 436, 437, 540, 581 and 613) (Table 2).

**Table 1:** *Pfdhfr and pfdhps *SNPs prevalence from *P.falciparum *parasites from Luanda, Angola.

**Gene**	**SNPs**	**Prevalence (%)**	**Gene**	**SNPs**	**Prevalence (%)**
	
***pfdhfr*** **(n = 61)**	16V/S	0/61 (0)	***pfdhps*** **(n = 30)**	436A	7/30 (23)
			
	50R	4/61 (6)		437G	2/30 (93)
			
	51I	57/61 (93)		540E	3/30 (10)
			
	59R	35/61 (57)		581G	2/30 (7)
			
	108N	61/61 (100)		613T/S	0/30 (0)
			
	140L	0/61 (0)			
			
	I164L	0/61 (0)			

**Table 2:** *Pfdhfr and pfdhps *haplotypes of *P.falciparum *parasites from Luanda, Angola.

Gene	Haplotypes	n	%	Mutated codons
***Pfdhfr*** **(n = 61)**	C**IRN**	31	51	3
	
	C**I**C**N**	21	34	2
	
	CN**RN**	4	6	2
	
	**RI**C**N**	4	6	3
	
	C**I**C **N**/S	1	2	1 or 2

***Pfdhps*** **(n = 30)**	S**G**KAA	18	60	1
	
	**AG**KAA	7	23	2
	
	SAKAA	2	7	0
	
	S**GE**AA	1	3	2
	
	S**GEG**A	2	7	3

Codon positions: *pfdhfr *C50R, N51I, C59R and S108N, *pfdhps *S436A/F/C, A437G, K540E, A581G and A613T/S. The sensitive haplotype is underlined and the mutated codons are shown in bold characters.

Only one sample presented a mixed infection for *pfdhfr*. Wild-type profiles were observed in *pfdhps *in two samples: one from Sambizanga and one from Imgombotas. No significant difference between the prevalence of the *pfdhfr *and *pfdhps *haplotypes was observed among the four municipalities.

## Discussion

This study documents a recent prevalence of *P. falciparum *antifolate resistance mutations in Luanda, the capital of Angola. It shows high levels of resistance-associated polymorphisms in the genes encoding the antifolate target enzymes *pfdhfr *and *pfdhps*, with most parasites containing 4 mutations that have been associated with an intermediate level of resistance, and 7% of them additionally containing *pfdhps *540E and 581G, both of which are associated with a higher level of resistance [10,20]. These molecular profiles are indicative of unsatisfactory response to SP and they are consistent with the results of a clinical trial in central Angola that showed poor antimalarial efficacy of this drug when it was used for treatment of children under five years old with uncomplicated malaria [21] as well as with the data of previous *in vivo *studies from East African countries [22-24].

The results showed a complex mixture of *pfdhfr/pfdhps *haplotypes in Luanda as already reported in other six provinces of Angola [25,26]. The 164L mutation was not disclosed, corroborating the findings of other nearby African countries [27-29]. The *pfdhfr *50R SNP herein found confers an increased level of resistance to pyrimethamine and this mutation is characteristic from South-American isolates [30] and, it was found only once in Africa, when Kenyan isolates were evaluated [31]. Three polymorphisms that mediate low-level resistance to SP, *pfdhfr *108N, *pfdhfr *51I, and *pfdhps *437G, were nearly universal and the *pfdhfr *59R mutation, which is associated with higher-level resistance, was common. Additional polymorphisms were also seen, notably the *pfdhps *540E and 581G mutations presented in the same sample that have been associated with a high-level of SP resistance.

Considering these data, it can be predicted that SP will have inadequate efficacy for the treatment of falciparum malaria, and further that it may no longer be appropriate for IPT in Angola. Indeed, although the number of samples available for study was limited, the prevalence of the *pfdhps *540E mutation might suggest that the cut-off level recently declared by the WHO for abandonment of IPT with SP in infants may be reached in this country. Unfortunately, no other drug offers simple, single-dose IPT. Surveillance works should provide advanced warning of SP failure in Angola.

## Conclusions

Since SP is still used for IPT in pregnant women as well as to treat bacterial infections, in Angola, a continuous pressure for such mutations selection may limit the therapeutic life span of this combination. Therefore the present data apparently argue against the use of this drug combination in Angola. The data also emphasize the need for a closer screening of these polymorphisms, especially *pfdhps *540E, along to the testing of other drugs for chemoprevention of malaria, including IPT in pregnant women and children, as an urgent priority.

## Competing interests

The authors declare that they have no competing interests.

## Authors' contributions

BEG participated in the design of the study, carried out the molecular analysis and drafted the manuscript; GALPC and FJILK were the responsible for blood samples collection; NKAO performed the PCR assays; FF helped in study design and field facilities; PJR and CTDR helped in the design of the study and reviewed the manuscript; MFFC conceived the study, coordinated its design, and finalized the manuscript. All authors have read and approved the final text.
